# Identification of Intermediate- to High-Risk Papillary Thyroid Carcinoma Patients Who May Be Safely Managed without the Performance of Delayed Stimulated Thyroglobulin Measurements following Total Thyroidectomy and Radioactive Iodine Therapy

**DOI:** 10.1155/2015/318916

**Published:** 2015-01-12

**Authors:** Kyung-Hee Kim, Min-Hee Kim, Ye-Jee Lim, Ihn Suk Lee, Ja-Seong Bae, Dong-Jun Lim, Ki Hyun Baek, Jong Min Lee, Moo-Il Kang, Bong-Yun Cha

**Affiliations:** ^1^Division of Endocrinology and Metabolism, Department of Internal Medicine, Seoul St. Mary's Hospital, College of Medicine, The Catholic University of Korea, No. 505 Banpo-Dong, Seocho-Gu, Seoul 137-701, Republic of Korea; ^2^Department of Surgery, Seoul St. Mary's Hospital, The Catholic University of Korea, Seoul 137-701, Republic of Korea

## Abstract

*Background*. The measurement of stimulated thyroglobulin (sTg) after total thyroidectomy and remnant radioactive iodine (RAI) ablation is the gold standard for monitoring disease status in patients with papillary thyroid carcinomas (PTCs). The aim of this study was to determine whether sTg measurement during follow-up can be avoided in intermediate- and high-risk PTC patients. *Methods*. A total of 346 patients with PTCs with an intermediate or high risk of recurrence were analysed. All of the patients underwent total thyroidectomy as well as remnant RAI ablation and sTg measurements. Preoperative and postoperative parameters were included in the analysis. *Results*. Among the preoperative parameters, age below 45 years and preoperative Tg above 19.4 ng/mL were significant risk factors for predicting detectable sTg during follow-up. Among the postoperative parameters, thyroid capsular invasion, lymph node metastasis, and ablative Tg above 2.9 ng/mL were independently correlated with a detectable sTg range. The combination of ablative Tg less than 2.9 ng/mL with pre- and postoperative independent risk factors for detectable sTg increased the negative predictive value for detectable sTg up to 98.5%. *Conclusions*. Based on pre- and postoperative parameters, a substantial proportion of patients with PTCs in the intermediate- and high-risk classes could avoid aggressive follow-up measures.

## 1. Introduction

In the absence of residual thyroid tissue, stimulated thyroglobulin (Tg) measurement 6–12 months after initial radioactive iodine (RAI) ablation is the most sensitive method for the early detection of persistent or recurrent disease [1]. Undetectable stimulated Tg (sTg) can predict complete disease remission with a very low recurrence rate (0.6–1.0%) [2]. Although it has an important role in long-term follow-up, sTg measurement is troublesome for patients because of the discomforts caused by thyroid hormone withdrawal (THW), such as cognitive dysfunction, physical and emotional discomfort, and impaired quality of life [3], and because of the economic burden of taking human recombinant TSH (rhTSH). Despite the inconvenience, follow-up with sTg is recommended for intermediate- and high-risk patients who undergo RAI ablation. Currently, there are insufficient data to support dispensing with sTg measurement after RAI ablation in intermediate- to high-risk patients, as shown in patients at low risk of recurrence [4].

Although there are many prognostic factors used to predict survival and recurrence, the predictive factors of a detectable sTg status after remnant RAI ablation are rarely investigated. Considering that repeated measurements of sTg are of limited value in patients who exhibit undetectable sTg at least once [4], it would be worthwhile to assess the risk of detectable sTg after initial treatment (total thyroidectomy and RAI remnant ablation). The identification of factors predicting detectable sTg after RAI ablation could guide clinicians in determining whether sTg should be measured in intermediate- to high-risk patients.

The aim of this study was to elucidate which clinical and pathologic parameters could predict detectable sTg after initial therapy in patients at intermediate to high risk of recurrence.

## 2. Methods

### 2.1. Patients and Study Designs

The records of consecutive series of 417 patients with PTC who underwent total thyroidectomy performed by a single surgeon (JS Bae) and had RAI ablation after surgery, from October 2008 through December 2009, at our institute were retrospectively collected. We excluded 47 patients who did not follow our protocol that is described below or who exhibited positive tests for thyroglobulin autoantibodies (>70 IU/mL) after the first RAI ablation or during the follow-up sTg after the first RAI ablation. Of the remaining patients, 27 patients with low-risk disease according to the American Thyroid Association (ATA) were excluded. Finally, a total of 346 patients with intermediate- to high-risk disease were included. Our protocol was as follows. At the time of total thyroidectomy, routine central lymph node dissection was performed. If lateral lymph node metastasis was suspected based on preoperative imaging studies, lateral lymph node dissection was performed. All of the subjects received remnant RAI ablation (3,700 MBq–5,550 MBq) at 2–4 months following surgical treatment under THW or through the use of rhTSH. THW was performed using T3 withdrawal for at least 2 weeks after switching from thyroxine to T3 for 2 weeks, and exogenously stimulated ablation was carried out by administering one injection of rhTSH (0.9 mg i.m., Thyrogen, Genzyme Corp., Cambridge, MA) on 2 successive days. Approximately 12 months later, follow-up examinations to determine disease status were performed using sTg measurements with or without radioiodine whole-body scans. Undetectable sTg and detectable sTg were defined as sTg < 1 ng/mL and sTg ≥ 1 ng/mL with negative thyroglobulin autoantibodies, respectively.

Patients' demographics, histopathological data, and laboratory findings were reviewed. The histopathologic parameters, including primary tumour size, multiplicity, thyroid capsular invasion, extrathyroidal extension, lymph node (LN) metastasis, and BRAF mutation status, were collected. The size of the tumour was recorded as the maximum diameter. The study protocol was approved by the ethical committee of the institutional review board of the Clinical Research Coordinating Center at Seoul St. Mary's Hospital.

### 2.2. Laboratory Measurements

Serum Tg was measured using an immunoradiometric assay (IRMA) kit (CIS Bio international, Cedex, France) with a functional sensitivity of 0.7 ng/mL. Anti-Tg Ab and anti-thyroid peroxidase Ab were measured using a competitive radioimmunoassay (RIA) kit (ZenTech, Angleur, Belgium); the upper normal values were 70 IU/mL and 50 IU/mL, respectively. The intra-assay and interassay coefficients of variation for both measurements are described in our previous study [5].

### 2.3. Statistics

Discrete data are summarised as numbers (percentages), and continuous data are expressed as means with standard deviations or median values and ranges, depending on the distribution. Two continuous variables were compared using the Mann-Whitney test or Student's *t*-test, and categorical variables were compared using the chi-squared test or Fisher's exact test where appropriate. *P* values less than 0.05 were considered statistically significant. Factors correlated with detectable sTg during follow-up were analysed using multivariate logistic regression according to the procedure “Enter.” Statistically significant variables based on univariate analysis (significance level: *P* ≤ 0.10) were included in a multivariate analysis using a binary logistic regression test. The cut-off values for preoperative Tg and stimulated Tg levels immediately prior to ablation (ablative Tg) used to predict the status of detectable sTg during follow-up were evaluated using receiver operative characteristic (ROC) curve analysis to determine the highest sum of sensitivity and specificity. All analyses were performed using SPSS for Windows, version 18.0 (Chicago, IL, USA).

## 3. Results

### 3.1. Baseline Characteristics of the Study Population

The median age of the patients at diagnosis was 45.89 years (18–74 years), and female patients comprised 81.8% (*n* = 283) of the population (Table 1). The mean tumour size was 11.6 ± 7.4 mm. Lymph node metastases were detected in 74.5% (*n* = 258) of the patients. According to the ATA risk classification, 62.1% (*n* = 215) of the patients belonged to the intermediate-risk group and 37.9% (*n* = 131) belonged to the high-risk group. Regarding the TSH stimulation method performed at the time of RAI ablation, THW was performed in 279 patients (80.6%) and rhTSH injections were administered in 67 patients (19.4%). The patient characteristics are summarised in detail in Table 1. No difference was determined between the THW group and rhTSH group with respect to ATA risk stratification or the rate of biochemical remission, as determined by undetectable sTg.

### 3.2. The Preoperative Parameters for Predicting Detectable sTg One Year after RAI Ablation

The proportion of patients older than 45 years in the undetectable sTg group was significantly higher than that in the detectable sTg group (57.6% versus 34.0%, *P* = 0.001) (Table 2). The median preoperative serum Tg was also significantly higher in the detectable sTg group than in the undetectable sTg group (33.60 ng/mL versus 12.62 ng/mL, *P* < 0.001). Preoperative Tg with a cut-off value of 19.4 ng/mL (area under the curve [AUC] 0.702) could predict detectable sTg with sensitivity, specificity, and positive and negative predictive values (PPV and NPV) of 66.0%, 63.8%, 24.3%, and 91.1%, respectively. Patients with preoperative Tg above the cut-off level comprise 41.6% of the study population (*n* = 144). The odds ratio (OR) of preoperative Tg with a cut-off value of 19.4 ng/mL for detectable sTg was 3.282 (95% confidence interval [CI]: 1.773 to 6.077, *P* < 0.001). In multivariate analysis with adjusting the clinical factors that included age, tumour size, and anti-TPO, the subjects with preoperative Tg greater than 19.4 ng/mL exhibited an increased risk of detectable sTg 6–12 months after RAI ablation (OR: 2.604, 95% CI: 1.339 to 5.062, *P* = 0.005).

### 3.3. The Postoperative Parameters for Predicting Detectable sTg One Year after RAI Ablation

Among histopathological parameters, the presence of thyroid capsular invasion and lymph node metastasis was significantly associated with detectable sTg (Table 3). Patients with central and lateral lymph node metastases exhibited an increased risk of detectable sTg compared with those with no lymph node metastasis (detectable sTg 18.5% in lymph node metastasis(+) versus detectable sTg 5.7% lymph node metastasis(−), *P* = 0.004). ATA risk stratification was also associated with detectable sTg. In contrast, the presence of thyroiditis was related to undetectable sTg. In addition to histopathologic factors, ablative Tg was analysed. With a cut-off value of 2.9 ng/mL (AUC 0.867), ablative Tg measurements were closely related to detectable sTg (sensitivity, 84.9%; specificity, 77.1%; PPV, 40.2%; NPV, 96.6%). In multivariate analysis, thyroid capsular invasion, lateral lymph node metastasis, and ablative Tg ≥ 2.9 ng/mL were independently correlated with detectable sTg (Table 3).

### 3.4. Identification of Patients Who May Be Safely Managed without the Performance of Delayed Stimulated Thyroglobulin Measurement after Radioactive Iodine Therapy

All but one of the patients with undetectable ablative Tg (≤1 ng/mL; *n* = 174, 50.3% of the population) exhibited undetectable sTg 6–12 months after initial therapies. The exceptional patient exhibited a sTg level of 1.07 ng/mL. This patient exhibited a 0.6 cm classical PTC with thyroid capsular invasion, extrathyroidal extension, and central lymph node metastasis (2 out of 8 evaluated nodules). During the follow-up period of 37 months, no identifiable lesion was noted using multiple imaging modalities (neck ultrasonography [US], chest computed tomography [CT], neck CT, WBS, and positron emission tomography CT scans) in this patient. Among the patients with ablative Tg under 2.9 ng/mL (*n* = 234, 67.6% of the total population), 96.6% (*n* = 226) exhibited sTg in the undetectable range. When preoperative and postoperative variables related to detectable sTg were combined to be analysed, the NPVs of ablative Tg < 2.9 ng/mL were 97.7% in the patients without thyroid capsular invasion, 98.5% in the patients without lymph node metastasis, and 97.5% in the patients with preoperative Tg < 19.4 ng/mL.

### 3.5. Recurrent Cases

Six out of 346 (1.7%) patients experienced clinical recurrence during the median follow-up of 40 months (30–59). All but one of the patients with clinical recurrence exhibited ablative Tg above 2.9 ng/mL and detectable sTg at 1 year after primary treatment. The patient who exhibited ablative Tg below the cut-off value of 2.9 ng/mL also exhibited lymph node metastasis and thyroid capsular invasion in pathologic reports, and sTg was detectable at follow-up. Recurrent lesions were limited to the cervical lymph nodes in four cases, and two cases exhibited distant lung metastasis.

## 4. Discussion

sTg measurement combined with neck US is strongly recommended for detecting persistent or recurrent disease in patients who have undergone total thyroidectomy followed by RAI ablation, especially those at intermediate to high risk [1, 6]. THW or rhTSH injection is required prior to sTg measurement, and it inevitably causes hypothyroidism and leads to high patient costs. Recent studies have demonstrated that postoperative sTg measurements performed before remnant RAI ablation could be useful in the decision to recommend remnant RAI ablation and further sTg measurements to determine disease status in low-risk patients [7, 8]. Recently, using the high sensitive Tg assay methods, of which the functional sensitivity is around 0.1 ng/mL, the need for measurement of sTg in follow-up after initial treatment may be reduced, especially in low-risk groups, because it has very high negative predictive value [9, 10]. In contrast, because no sufficient data exist yet to justify the omission of sTg measurements in intermediate- to high-risk patients, patients are subjected to the inconvenience of sTg measurements. Based on our study's results, we suggest that a substantial number of patients in these groups could avoid sTg measurement.

In our analysis, more patients younger than 45 years belonged to the detectable sTg group. It has been reported that lymph node metastases are more frequently detected in adolescents and young adults than in older patients [11, 12]. Consistent with previous studies, we observed that patients younger than 45 years exhibited significantly higher N stages than older patients (N0, *n* = 22 (13.8%); N1a, *n* = 111 (69.8%); N1b, *n* = 26 (16.4%) in patients < 45 years and N0, *n* = 66 (35.3%); N1a, *n* = 95 (27.5%); N1b, *n* = 26 (13.9%) in patients ≥ 45 years; *P* < 0.001). Given that the lymph node metastasis status was an important independent risk factor for predicting detectable sTg, the significance of age may be a result of the relationship between age and lymph node metastasis. Furthermore, among preoperative parameters, serum Tg levels could be a possible marker for predicting detectable sTg in intermediate- or high-risk patients. Several studies have demonstrated that preoperative Tg could be a surrogate for malignant nodules when determining the need for a cytologic examination of indeterminate nodules [13, 14]. However, only one study has evaluated the role of preoperative Tg as a prognostic marker for PTC, although the study failed to support its use [15]. However, this study included a small number of subjects (*n* = 71). Though further evaluation to explain and validate the results of preoperative Tg is required, our results could suggest that preoperative Tg may play a role in determining whether sTg should be followed or not in patients reluctant to undergo sTg measurement.

Previous studies have demonstrated that the tumour size, the presence of lymph node metastases, the TNM stage, extrathyroidal extension, thyroid capsular invasion, and tumour multiplicity in operative findings were important clinical and histological factors for the prognosis of PTC [16–22]. Molecular markers such as galectin-3 or BRAF mutation have also been suggested as prognostic factors [23–25]. Unfortunately, none of those studies demonstrated that pathological findings could be used in clinical practice to guide decisions about whether to perform follow-up sTg measurements. In this study, among the postoperative parameters for predicting detectable sTg, the presence of thyroid capsular invasion and lymph node metastasis and the absence of thyroiditis were risk factors in univariate analysis. In particular, the presence of thyroid capsular invasion and lymph node metastasis was identified as the independent risk factors for detecting sTg 1 year after the initial treatment.

Ablative Tg was also identified as a prognostic factor in previous studies [26–30]. Lee et al. [28] demonstrated that a cut-off ablative Tg level of 2 ng/mL exhibited a high NPV (94.9%) with respect to tumour recurrence. Another study found that the NPV of ablative Tg < 1 ng/mL or < 10 ng/mL combined with negative ultrasonography was 100% in patients with a low risk of recurrence [31]. In addition, an increased risk of ablation failure was observed when ablative Tg was 5 ng/mL or more [30]. However, considering that the surgical extent could influence the level of ablative Tg [32] and that several studies have reported that the outcome of cancer surgery is influenced by surgeon training [8, 33], our study was superior to others in that our population was very homogenous in terms of routine central node dissection and operation by a single surgeon. Additionally, the role of ablative Tg combined with clinicopathological parameters was evaluated, focusing on decisions about follow-up strategies.

**Figure 1:**
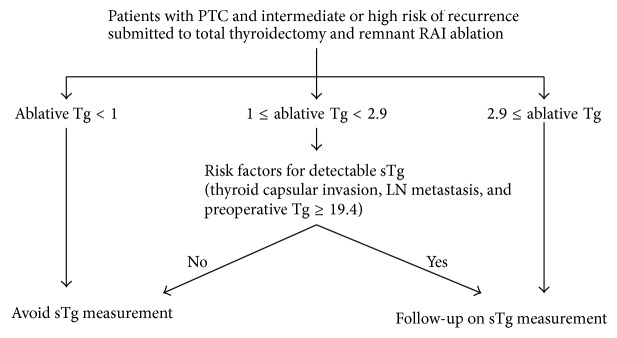
An algorithm for determining whether to measure sTg in thyroid cancer patients at intermediate to high risk of recurrence at 6–12 months after radioactive remnant ablation.

Using a cut-off value of undetectable ablative Tg (<1 ng/mL), the NPV reached nearly 100%. We therefore suggest that sTg measurement 1 year after initial treatment could be avoided in the majority of patients whose ablative Tg levels were undetectable. The NPV of the optimal cut-off value of 2.9 ng/mL, which was 96.6% in the evaluation of all study population, increased to 97.7% when patients with thyroid capsular invasion were excluded, to 98.5% when patients with LN metastasis were excluded, and to 97.5% when patients with preoperative Tg ≥ 19.4 ng/mL were excluded. As Figure 1 shows, it could be suggested that the lymph node metastasis status, thyroid capsular invasion, and preoperative Tg levels in combination with a certain cut-off level of ablative Tg can assist clinical decision-making about whether to perform follow-up sTg measurements after remnant RAI ablation in patients at intermediate and high risk of recurrence. In our study, there were 60 patients with ablative Tg level of 1 ng/mL or above but less than 2.9 ng/mL. In that group of patients, 5 patients did not have any risk factors for detectable sTg and none of them showed detectable range of sTg in the follow-up. On the contrary, among 55 patients with one or more risk factors for detectable sTg, 6 patients (11%) had been revealed to have detectable sTg. Therefore, patients with 1 ng/mL ≤ sTg < 2.9 ng/mL, who do not possess any risk factors, could avoid measurement of sTg in follow-up as shown in Figure 1. Most cases of clinical recurrence in PTC patients are limited to cervical locoregional disease; in those cases, neck US is the most sensitive and practical modality for detection [4, 34]. In this context, if the patients, even those who are at intermediate to high risk, do not possess any independent risk factors for detectable sTg, US and serial follow-up of Tg measurement under TSH suppression would be reasonable and sufficient for surveillance; sTg would not be necessary. It could be expected that the number of patients who would require sTg measurements would decrease using this approach.

The present study has several limitations. First, our investigation was based on retrospective data. Second, the median follow-up duration was 40 months, which is somewhat short for evaluating disease status in PTC. An additional limitation is that the primary outcome of the study was not the recurrence of disease. However, considering that the sTg value measured during the follow-up of PTC after near-total or total thyroidectomy and initial remnant RAI ablation is well recognised as a highly predictive method for persistent cervical disease or distant metastases [1, 35, 36], detectable sTg as a primary outcome is still worthwhile. Last, the study population consisted of both THW (80.6%) and rhTSH (19.4%) groups at RAI ablation. A previous study presented questions about the predictive value of serum Tg levels for disease status in patients who underwent rhTSH stimulation upon remnant RAI ablation compared with those who were stimulated with THW because ablative Tg under rhTSH stimulation is measured 48 hours after RAI ablation and could cause radioiodine-induced thyroid cell damage and Tg release [7]. Contrary to this concern, the NPV of ablative Tg levels obtained via rhTSH injections for predicting persistent or recurrent disease at one year after initial therapies was similar to the NPV of ablative Tg levels obtained via THW [12]. Our study also demonstrates that a cut-off level of 2.9 ng/mL was the best for optimal sensitivity and specificity using ROC curves for serum Tg stimulated with rhTSH upon remnant RAI ablation (data not shown), and the NPV with ablative Tg stimulated with rhTSH was higher than the NPV in cases stimulated with THW (100% versus 96%, resp.).

In conclusion, lymph node metastasis, thyroid capsular invasion, high preoperative Tg, and ablative Tg were independent prognostic factors for predicting detectable sTg in follow-up studies after total thyroidectomy and remnant RAI ablation treatment. Even in patients who are considered intermediate to high risk based on ablative Tg in combination with other clinical risk factors, the possible omission of sTg measurements should be considered when making decisions about follow-up strategies.

## Figures and Tables

**Table 1:** Baseline characteristics of patients.

Parameters	*N* = 346
Age at diagnosis (years)^a^	45.89 (18–74)
Female gender	283 (81.8%)
Interval from surgery to 1st RAI ablation (months)^b^	3.81 ± 1.17
Interval from 1st RAI ablation to follow-up sTg (months)^b^	12.77 ± 3.23
Median follow-up duration (months)	38.9 (11.6–59.7)
Recurrent cases	6 (1.7%)
Tumour size (mm)	
≤10	186 (53.8%)
10–40	157 (45.4%)
40≤	3 (0.9%)
Thyroid capsular invasion	287 (82.9%)
Extrathyroidal extension	282 (81.5%)
Cervical lymph node metastasis	258 (74.5%)
N0	88 (25.4%)
N1a	206 (59.5%)
N1b	52 (15.0%)
BRAF mutation/evaluation^c^	284/336 (84.5%)
Thyroiditis	58 (16.8%)
Multiplicity	
None	206 (59.5%)
One lobe	52 (15%)
Both lobes	87 (25.1%)
ATA risk classification	
Intermediate	215 (62.1%)
High	131 (37.9%)
Method of TSH stimulation at RAI ablation	
Thyroid hormone withdrawal	279 (80.6%)
rh TSH administration	67 (19.4%)

^a^Median (range).

^b^Mean ± standard deviation.

^c^Number of patients who were examined for BRAF mutation of tumor.

**Table 2:** Preoperative factors for predicting detectable sTg in the 1-year follow-up.

Parameters	Undetectable sTg (*n* = 293, 84.7%)	Detectable sTg (*n* = 53, 15.3%)	*P* value
Age (≥45), *n* (%)	169 (57.6%)	18 (34.0%)	0.001
Male, *n* (%)	54 (18.4%)	9 (17.0%)	0.801
Free T4 (ng/mL), mean (range)	1.27 (0.75–2.31)	1.34 (0.91–2.96)	0.248
TSH (mIU/L), mean (range)	1.90 (0.03–182.00)	1.81 (0.07–6.83)	0.852
Anti-TPO Ab (IU/mL), *n* (%)	42 (14.3%)	3 (5.6%)	0.085
Anti-Tg Ab (IU/mL), *n* (%)	55 (18.8%)	6 (11.3%)	0.190
Tg (ng/mL), median (range)	12.62 (0.05–405.40)	33.60 (0.74–696.52)	<0.001
19.4>, *n* (%)	184 (91.1%)	18 (8.9%)	<0.001
19.4≤, *n* (%)	109 (75.7%)	35 (24.3%)	

**Table 3:** Postoperative parameters for predicting detectable sTg.

Parameters	Univariate analysis			Multivariate analysis		
	Odds ratio	95% CI	*P* value	Odds ratio	95% CI	*P* value
Tumour size						
≤10	1 (ref.)					
10–40	1.606	0.886–2.909	0.118			
40≤	3.543	0.309–40.648	0.309			
Thyroid capsular invasion	3.776	1.137–12.542	0.030	4.293	1.086–16.968	0.038
Extrathyroidal extension	1.329	0.593–2.977	0.489			
Lymph node metastasis						
N0	1 (ref.)			1 (ref.)		
N1a	2.529	1.014–6.306	0.047	2.926	0.973–8.802	0.056
N1b	7.250	2.636–19.938	<0.001	7.495	2.094–26.832	0.002
ATA						
Intermediate	1 (ref.)			1 (ref.)		
High	1.724	0.957–3.108	0.070	1.222	0.538–2.777	0.632
BRAF mutation	0.806	0.365–1.778	0.593			
Thyroiditis	0.228	0.054–0.971	0.046	0.327	0.062–1.714	0.186
Multiplicity	0.968	0.532–1.760	0.914			
Ablative Tg						
2.9>	1 (ref.)			1 (ref.)		
2.9≤	18.974	8.526–42.224	<0.001	18.433	7.818–43.458	<0.001
